# Modelling Malpighian tubule crystals within the predatory soil mite *Pergamasus longicornis* (Mesostigmata: Parasitidae)

**DOI:** 10.1007/s10493-017-0137-7

**Published:** 2017-05-24

**Authors:** Clive E. Bowman

**Affiliations:** Mathematical Institute, University of Oxford, Oxford, OX2 6GG UK

**Keywords:** Bayes, Digestion, Egestion, Excretion, Input-Output, Kinetics, Proteresis, Pulse-chase

## Abstract

The occurrence of refractive crystals (*aka* guanine) is characterised in the Malpighian tubules of the free-living predatory parasitiform soil mite *Pergamasus longicornis* (Berlese) from a temporal series of histological sections during and after feeding on larval dipteran prey. The tubular system behaves as a single uniform entity during digestion. Malpighian mechanisms are not the ‘concentrative’ mechanism sought for the early stasis in gut size during the second later phase of prey feeding. Nor are Malpighian changes associated with the time of ‘anal dabbing’ during feeding. Peak gut expansion precedes peak Malpighian tubule guanine crystal occurrence in a hysteretic manner. There is no evidence of Malpighian tubule expansion by fluid alone. Crystals are not found during the slow phase of liquidised prey digestion. Malpighian tubules do not appear to be osmoregulatory. Malpighian guanine is only observed 48 h to 10 days after the commencement of feeding. Post digestion guanine crystal levels in the expanded Malpighian tubules are high—peaking as a pulse 5 days after the start of feeding (i.e. after the gut is void of food at 52.5 h). The half-life of guanine elimination from the tubules is 53 h. Evidence for a physiological input cascade is found—the effective half-life of guanine appearance in the Malpighian tubules being 7.8–16.7 h. Crystals are found present at all times in the lumen of the rectal vesicle and not anywhere else lumenally in the gut at all. No guanine was observed inside gut cells. There is no evidence for the storage in the rectal vesicle of a ‘pulse’ of Malpighian excretory products from a discrete ‘pulse’ of prey ingestion. A latent egestive common catabolic phase in the gut is inferred commencing 12.5 h after the start of feeding which may cause the rectal vesicle to expand due to the catabolism of current or previous meals. Malpighian tubules swell as the gut contracts in size over time post-prandially. There is evidence that at a gross level the contents of the rectal vesicle are mechanically voided by the physical mechanism of overall gut expansion altering the effective idiosomal volume available during prey ingestion. A complete cycle of feeding, digestion, egestion and excretion is approximately 9 days. Hunger/starvation likely commences at 10 days after the start of feeding. Up to 15 days may be needed to completely clear the idiosoma of excretory material. Nomograms for predicting the likely feeding time of mites from observations of idiosomal guanine in field samples indicate that as few as 5–6 mites scoring positive for Malpighian tubule guanine out of 20 infers a high probability that the typical time from start of feeding in a population sample was about 6 days (range 3–8 days) ago.

## Introduction

**Fig. 1:**
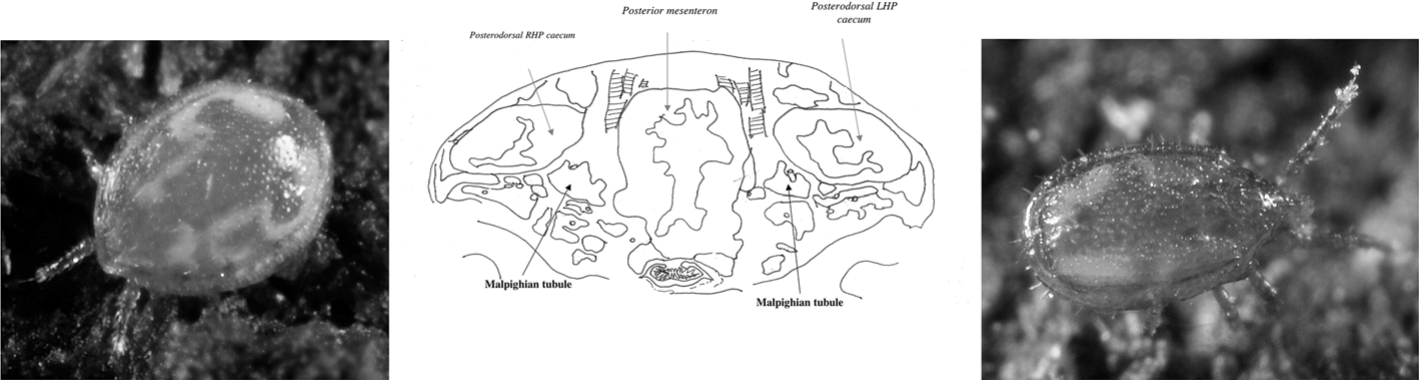
Illustrative Malpighian structures in the body of typical soil mesostigmatids. NB In adult *Pergamasus* spp. the integument is too opaque to *easily* photograph guanine deposits through it. *Left* *Uropoda* sp.? Norfolk, Virginia, USA November 27, 2008; from a colour photograph © Scott Justis 2008 with permission, showing a pair of elongate convoluted tubular white structures under the length of the idiosomal cuticle. *Middle* Sectional drawing of posterior section of idiosoma in female *Pergamasus longicornis* 18 h after start of feeding showing Malpighian tubules nestling between and strongly associated with gut regions. *Right* *Trachytes* sp.? Norfolk, Virginia, USA December 16, 2008; from a colour photograph © Scott Justis 2008 with permission, showing a pair of expanded pale posterolateral deposits inside the idiosoma

In a carnivore, food must be ingested and usually digested before it can be assimilated. Broadly speaking for a predatory acarine, prey can be seen as a single ‘gulp’ of protein to be handled metabolically. A part of protein catabolism is the production of various nitrogenous waste products that need excretion to maintain physiological homeostasis. Different metazoans use different depots to sequester this nitrogen including ammonia, urea, uric acid (often as urates), xanthine and guanine (Rastogi 1971). Mites are arachnids. For more than a century, the purine base guanine has been known to accumulate in spiders (see Fausek 1909; Gorup-Besanez and Will 1849), comprising 70–85% of the nitrogen excreted in spiders (see Atkinson and Chorlton 1956). A different purine, xanthine is found in xiphosaurans and is particularly high in some desert scorpions (Polis 1990; Yokota and Shoemaker 1981). Guanine is found in appreciable amounts within the excreta of other scorpions (Haggag and Fouad 1965), amblypygids, uropygids and solifugids (Rao and Gopalkrishnareddy 1962). It is biosynthesised in ticks (Bassal 1977; Hamdy and Sidrak 1982) forming in their Malpighian tubules (Guirgis 1971; Hamdy 1972) and excreted in large quantities (Kitaoka 1961). Visually such accumulates are white, and appear refractive under transmitted light. Amongst these chelicerates, most mites are no different. Many photographs of soil inhabiting mesostigmatids show such white tubular deposits internally (see Fig. 1). Similar sub-cuticular refractive crystals have been described during feeding in *Pergamasus longicornis* (Berlese) by Bowman (1984) and ascribed to guanine in the Malpighian tubules. Guanine and Malpighian tubules are claimed not to exist in phthiracarids (Dinsdale 1975).

**Fig. 2:**
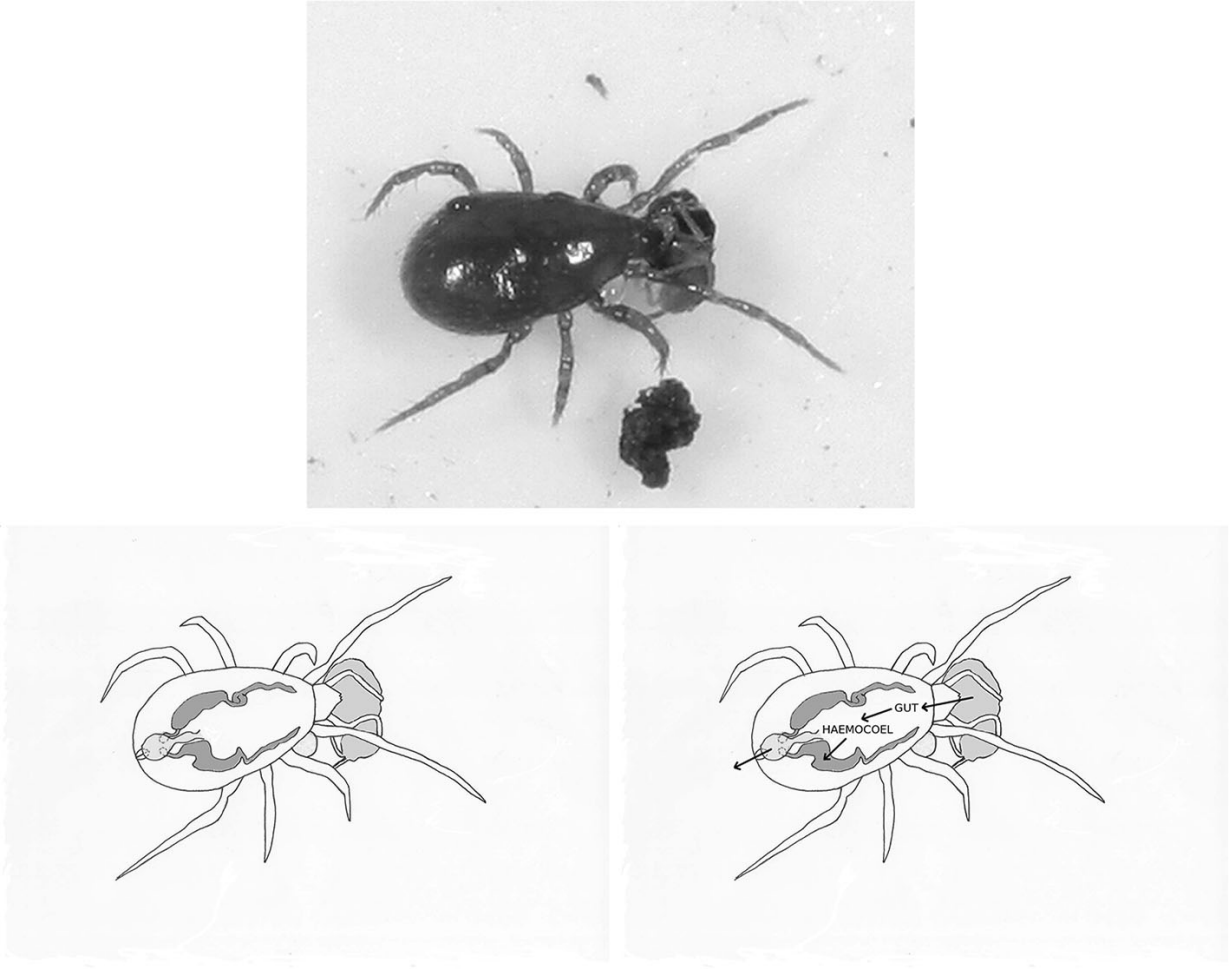
*Upper* Pergamasid feeding on globular springtail (from a colour photograph by Tom Murray 2006, © with permission). Note the mite’s almost complete encasement in an opaque chitinous ‘suit of armour’. *Lower* Overlain schematics of *Pergamasus longicornis* Malpighian tubules (*dark grey*); springtail (*pale grey to the right*); swollen rectal vesicle and (forward of it) the tubular hind gut (both *pale grey shading* to the posterior of the idiosoma); together with coxal droplet (off-white under coxa I—see Bowman 1984). Fore- and mid-gut omitted for clarity (see Bowman 2014 for diagram). *Left* Malpighian tubules arise anteriorly and ventrally under the anterodorsal caeca—*here they are drawn as ‘contracted’*. They are closed at their anterior ends. They then rise and enlarge—*here they are drawn as ‘expanded’*—to sit around the posterior mesenteron. Then they fall, to debouch ventrally into the rectal vesicle via a narrow entrance. The rectal vesicle opens into the pygidial-valved anus ventrally. *Right* Schematic arrows show fluidised prey enters the gut, is broken down and transported into the haemocoel. Excretory products are removed from the haemocoel into the Malpighian tubules and voided via the rectal vesicle. Faecal material from the fore- and mid-gut (if produced) would flow via the hind-gut into the rectal vesicle

Whilst a variety of publications over the years describe the structure of Malpighian tubules in various acarines (cf. Balashov and Raikhel 1973; Coons and Axtell 1971; Sonenshine 1970), the excretory system in general remains poorly known for mesostigmatid mites. Neumann (1941) describes the complete internal anatomy and histology of *Parasitus kempersi* Oudemans (Parasitidae) including the excretory organs and nephrocytes but not how they change over time. Both purines (xanthine and guanine) are poorly soluble, so nitrogen can be excreted without much water loss—a useful trick for soil arthropods (see Little 1983). It is important in understanding such animals’ adaptations, not just to know how an organ or tissue is structured but also how it works. Bowman (2014) pointed out that an understanding of the temporal changes in the status of the Malpighian tubules and the time course of the production of waste material would help confirm or refute explanations of the ‘stiff’ digestive mechanisms in the gut of *P. longicornis* offered in his paper. The coupling of excretion to feeding is documented in argasids (Hamdy 1973)—a sister group with holothyrids and ixodids to the mesostigmatids. Mesostigmatid excretory physiology is not known in great detail but is assumed to be dependant upon digestive processes in some way too. The temporal concept of this paper is that pergamasid excretion should follow after or in part contemporaneously with the digestion of a meal. Any fluidised material absorbed by the gut being transformed in the haemocoel into excretory products and dealt with as in Fig. 2. Any food-derived digestive remnants arising out of the fore- and mid-gut also being voided via the rectal vesicle.

By virtue of their year-round occurrence, their intermittent bolus feeding and their long life-cycle, pergamasids are good model species for soil predatory acarines. Their large size is also an advantage in making physiological investigations as feasible as possible. In his scheme explaining pergamasid gut size changes during digestion, Bowman (2014) proposed three certain key phenomena regarding the stimulation of excretion by feeding. Firstly, that since the size of the rectal vesicle is $180^{\circ }$ out of phase with the other gut parts (i.e. being full when they are empty, or, being empty when they are full), it is simply a passive storage vessel before gut waste products and the excretory materials from the Malpighian tubules, which debouch into it, are voided. Secondly, that metabolism should produce excretory products *throughout* digestion, and that post digestion levels of excretory product should be high. Finally, that Malpighian fluid excretion may be a physical water balancing mechanism during feeding.

This paper, based upon detailed serial histological data scored from a time series of *P. longicornis* at various points over 2 weeks from the onset of feeding, seeks to describe when excretory products (crystalline guanine) are present during feeding and digestion. The experimental concept is that observing Malpighian tubules expanded with crystals indicates high guanine levels, observing them contracted or acrystalline indicates low levels. Furthermore that living in a ‘suit of armour’ constrains the independence of organ size and behaviour given likely digestive and metabolic processes when feeding by this mite. In other words physicality informs physiology, and physiology informs physicality.

The underlying analytic concept of this paper is that simple first-order differential equations can effectively describe the mite’s physiology. As such kinetic ‘input-output’ modelling of changes will inform the inference of what metabolic processes are happening. A variety of pre-specified questions or hypotheses are addressed. This paper first checks whether the state of expansion/contraction in Malpighian tubules is different anteriorly from that posteriorly (as is the case for the gut—see Bowman 2014). Then, examines the three key points given above to look for supportive or contra-evidence. The suggested physiological scheme and its assertions are critically tested as follows:

- Is there evidence of Malpighian expansion with fluid during the ‘concentrative’ phase of pergamasid feeding (i.e. 10 to 90 min from start of feeding)?
- Is crystalline guanine present during the early ‘anal dabbing’ phase (i.e. <1.5 h after start of feeding)?
- Is the time course of guanine production congruent with a slow digestion phase predominating after 120 min from the start of feeding?
- Are excretory products only produced in the later phase after the mite has stopped feeding?
- Does the time course of guanine production match the estimated total digestion time (of 52.5 h)?
- Are guanine crystals mechanically voided from the body by the physical mechanism of overall gut size changes?

Answering these in particular will validate or refute his hypotheses.

However, more can be done using this model mesostigmatid. For instance:

- What can be said of any coupling of Malpighian guanine to gut changes using simple kinetic models?
- Does the excretory system too exhibit ‘stiff’ behaviour?
- Can these physiological insights be applied to field ecology?

Critical explanation of the observed Malpighian tubule changes is offered together with suggestions to confirm or refute the physiological results and any mechanisms involved experimentally are given.

## Materials and methods

Mites were collected by hand from leaf litter sampled at a variety of deciduous woodland sites in Merseyside and Hertfordshire, UK in 1977. Mites were kept individually at room temperature and >90% rh throughout. Mites were starved for 1 week and then fed one final instar larva of the fruit fly *Drosophila melanogaster *(vestigial wing strain). At 28 distinct pre-specified log-spaced elapsed times from the commencement of feeding, a total of thirty four mites were destructively fixed in cold Susa, dehydrated through graded isopropyl alcohol, into xylene and double embedded in celloidin and paraffin wax. Sections were taken at 7$\upmu$, stained with Mallory’s Triple Stain and mounted in DePeX. The Malpighian tubules associated with each of the 15 gut regions were scored as: contracted and containing no guanine (0); containing some (1) guanine; or, expanded with lots of guanine (2). All gut regions (see Bowman 2014) including the rectal vesicle were also scored (0 = none, 1 = some, 2 = lots) for the presence of guanine within their lumen. Refractive material comprising of large crystals under Nomarski phase contrast interference light microscopy was taken to indicate the presence of guanine. No chemical confirmation of the presence of this purine was undertaken. Relative occurrence is assumed to measure relative amounts. Amounts were not normalised by gut volume to derive a surrogate of concentration. Due to collecting constraints, no distinction was made between male (elapsed time after the start of feeding: 0 min, 2, 5, 5, 10, 25, 60, 90 min, 6 h, 12, 48, 96, 168, 192, 240, 288 h) and female (0 min, 15, 20, 20, 30, 30, 60, 90 min, 2 h, 4, 8, 18, 24, 72, 120, 144, 216, 336 h) mites. Genders are pooled as in Bowman (2014). All data were coded, stored and manipulated in Access97, Excel, or SAS6.12. Diagrams were produced in SAS 6.12, Excel, Powerpoint and GIMP. Elapsed time from zero was log transformed (time > 0). A logarithmic scale for elapsed time was chosen upon the simplest assumption that physiological changes represent first order rate processes (Lister et al. 1988)—this is the same assumption as in the models of Sabelis (1990). Natural ($\hbox {log}_{e}$) logarithms were used throughout.

Bayesian modelling of the probability of Malpighian guanine (*M*) used OpenBugs 3.23 with non-informative Jeffreys priors and a logit link function where appropriate (Congdon 2001; McCullagh and Nelder 1989). A burn-in of 30,000 updates followed by summaries of a further 30,000 updates was used. The notation ‘hat’ is used to mean ‘estimated value’. The log odds ratio comparing anterior sections (subscript = 1) to posterior sections (subscript = 2) was calculated as $ln[\frac{\pi _1}{(1-\pi _1)}\cdot \frac{(1-\pi _2)}{\pi _2}]$ where $\pi$ is the estimated probability of the occurrence of guanine in the Malpighian tubules. This statistic is asymptotically Normally distributed as $N(ln(odds\ ratio),\sigma ^{2}_{ln(odds\ ratio)})$ under the null of no distinction between the two regions. Posterior predictive distributions (Aitchison and Dunsmore 1975) of the likely time since the start of feeding for any new observation (or observed proportions) used a uniform prior for group membership.

Kinetic modelling of the Bayesian posterior mean estimates was done by exponential stripping (Kirkup and Sutherland 1988) on an arithmetic time scale using Excel and R. A first order differential empirical model for the probability of guanine occurrence at any time *t* in the observed ‘current’ Malpighian compartment was used via the *directed* output-input relationship:

$$\begin{aligned} M[t]=a_{el}.e^{b_{el}.t}-a_{in}.e^{b_{in}.t} \end{aligned}$$

where: *M* denotes the probability of guanine [observed at time *t*], the subscript *in* denotes input, and the subscript *el* denotes elimination (output). For the rate constant $b_{el}<0$ the effective size of $a_{el}$ declines away with a half-life $t_{\frac{1}{2}}=\frac{ln(2)}{b_{el}}$. If there was no input then $a_{el}=M[0]$. The stripping process traditionally fits late occurring data under the assumption that elimination is dominating and that absorption has essentially completed. Then subtracts the fitted values (assuming this elimination) from the original data to form adjusted data. Then fits the early occurring adjusted data when absorption (i.e. input) is dominating and elimination only just under-way in order to estimate the absorption parameters. So, the contribution $a_{in}.e^{b_{in}.t}$ represents in a positive sense the first order ‘elimination’ ($b_{in}<0$) from a notional previous or up-stream compartment being now input into the observed down-stream Malpighian one. The approach produces a smooth knot or continuity between the two processes. The underlying differential equation is $\frac{dM}{dt}=c.H-v.M$ where *c* represents catabolism, *v* represents voiding and *H* represents the notional previous compartment (the haemocoel). The original ingestive pulse of prey (whether as a true bolus, or equivalent to a constant rate infusion infusion, or a first-order process) is considered so far away earlier that the effect of its shape can be considered as negligible and solely captured by the dynamic behaviour of the notional compartment in advance of the Malpighian tubules. Assimilation is assumed to already have reached its asymptotic state.

In this example $\hat{a}_{in}=\hat{a}_{el}=e^{(max[\hat{M}_{t}])}$ since stripping should ensure a crossing or knot point for the functions at the peak of *M*[*t*]. The half-life of the input function is $\frac{ln(2)}{b_{in}}$. All physiological phenomena are assumed to be present from $t=0$ after the start of feeding, so all other matters being equal, the linear superposition principle ensures that the shape of any profile from compartment to compartment spreads out as the signal passes along a series of compartments arranged one-after-another. The initial signal thus becomes ‘blurred’ and attenuated (*per force* diminished in extent)—even if an interlinked network of compartments are assumed. Such models as this can be re-expressed into equilibrium partition models to derive mechanistic volumes of distribution, clearances etc. Here for instance

$$\begin{aligned} max[\hat{M}_{t}]=\hat{M}_{t_{max}}=\left( \frac{D}{V}\right) \cdot \frac{\hat{b}_{in}}{\hat{b}_{in}-\hat{b}_{el}}\cdot (e^{\hat{b}_{el}.t_{max}}-e^{\hat{b}_{in}.t_{max}}) \end{aligned}$$

where $(\frac{D}{V})$ is an estimated proportionality constant (*aka* the ingested ‘dose’ of food over the volume of distribution of the system) and $t_{max}$ is the time of the fitted maximum probability of Malpighian guanine.

## Results

**Fig. 3:**
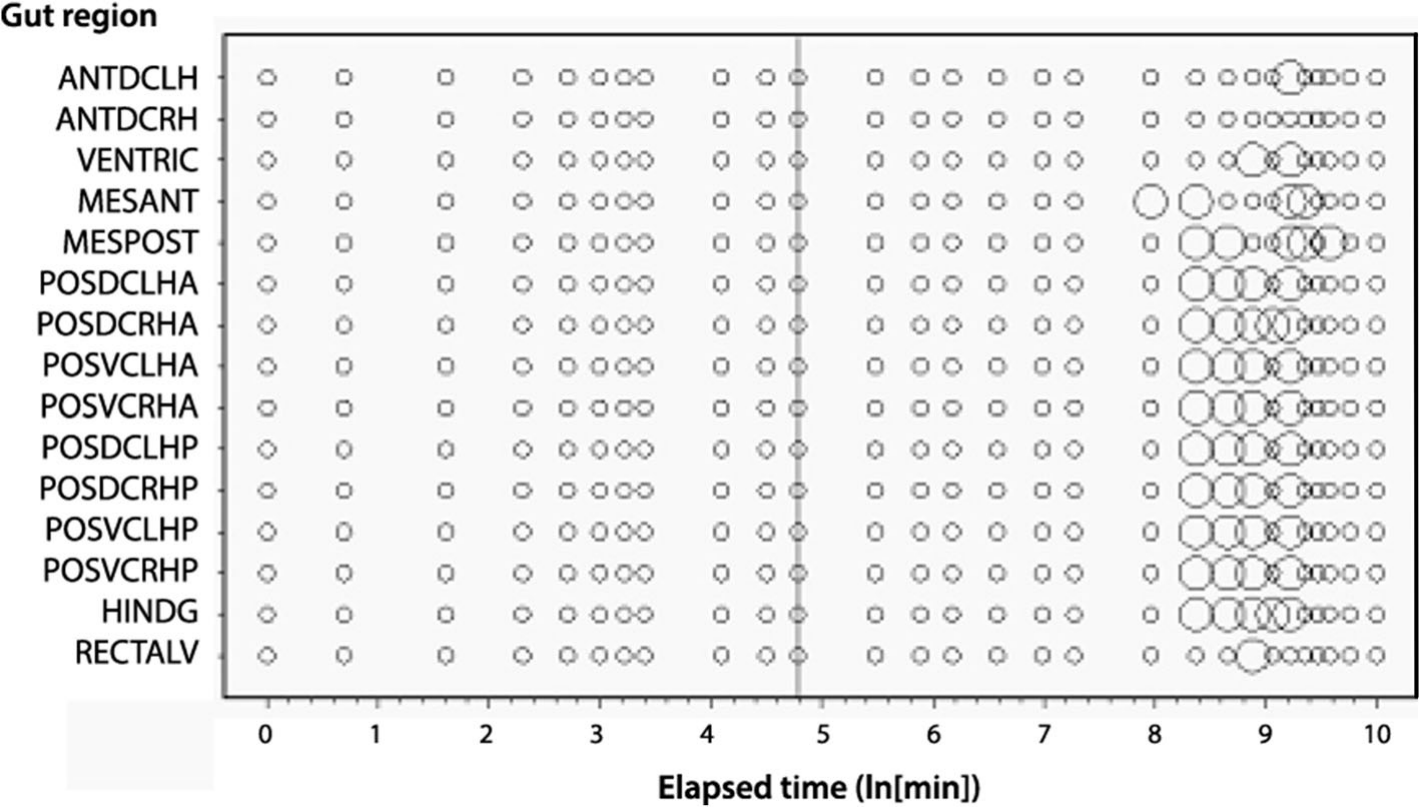
Schematic of *Pergamasus longicornis* observed Malpighian tubule status associated with each gut region (ordered anterior to posterior) as: Expanded with lots of guanine (*large circles*), or, Contracted without guanine (*small dots*). Intermediate size circles represent an intermediate score averaged over mite replicates. Time is from the commencement of feeding and is on a natural logarithmic scale. *Grey line* is at the 120 min optimal knot position between net gut-filling and net gut-emptying (see Bowman 2014). Associated gut region abbreviations: Anterodorsal caecum LH = ANTDCLH; Anterodorsal caecum RH = ANTDCRH; Ventriculus = VENTRIC; Mesenteron anterior = MESANT; Mesenteron posterior = MESPOST; Posterodorsal caecum LH anterior = POSDCLHA; Posterodorsal caecum RH anterior = POSDCRHA; Posteroventral caecum LH anterior = POSVCLHA; Posteroventral caecum RH anterior = POSVCRHA; Posterodorsal caecum LH posterior = POSDCLHP; Posterodorsal caecum RH posterior = POSDCRHP; Posteroventral caecum LH posterior = POSVCLHP; Posteroventral caecum RH posterior = POSVCRHP; Hind gut = HINDG; Rectal vesicle = RECTALV

Scoring for the expansion of, and the presence of guanine in, the Malpighian tubules associated with each gut region is presented in Fig. 3. Figure 7 displays the results of the observed Malpighian tubule status scored in this study averaged over all gut regions (bar the rectal vesicle) against the backdrop of overall gut size changes described by Bowman (2014). Measuring gut expansion/contraction is not a good surrogate of *all* aspects of the digestive and excretory physiological status in this carnivorous poikilotherm.

**Fig. 4:**
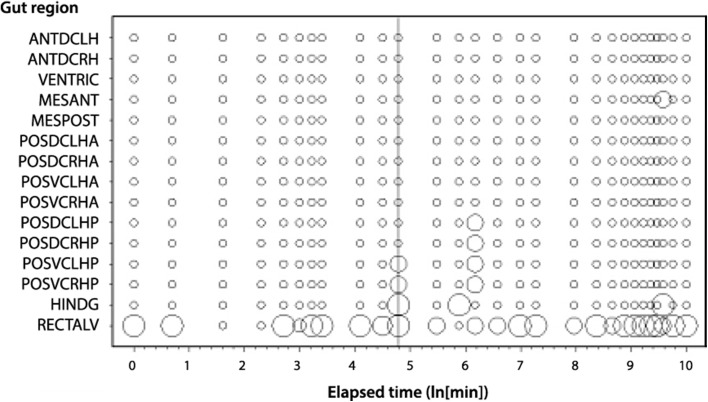
Schematic of *Pergamasus longicornis* observed presence of lumenal guanine crystals in the *gut* for each gut region (ordered anterior to posterior) as: Lots of lumenal guanine (*large circles*), or, Without lumenal guanine (*small dots*). Intermediate size circles represent an intermediate score averaged over mite replicates. Time is from the commencement of feeding and is on a natural logarithmic scale. *Grey line* is at the 120 min optimal knot position between net gut-filling and net gut-emptying (see Bowman 2014)

Scoring for the presence of guanine in the lumen of each of the gut regions is presented in Fig. 4. With very few exceptions (that by their distribution over the individual sections inspected in detail were probably due to rare *ad hoc* back reflux from the rectal vesicle on fixing or inadvertent cross-contamination when sectioning)—guanine was found in substantial quantities only in the lumen of the rectal vesicle, as in ticks (Hamdy and Sidrak 1982). Essentially guanine is found nowhere else in the lumen of the mesostigmatid fore-, mid- or hind-gut in this study. Never was any guanine noted intracellularly under light microscopy of the gut.

**Fig. 5:**
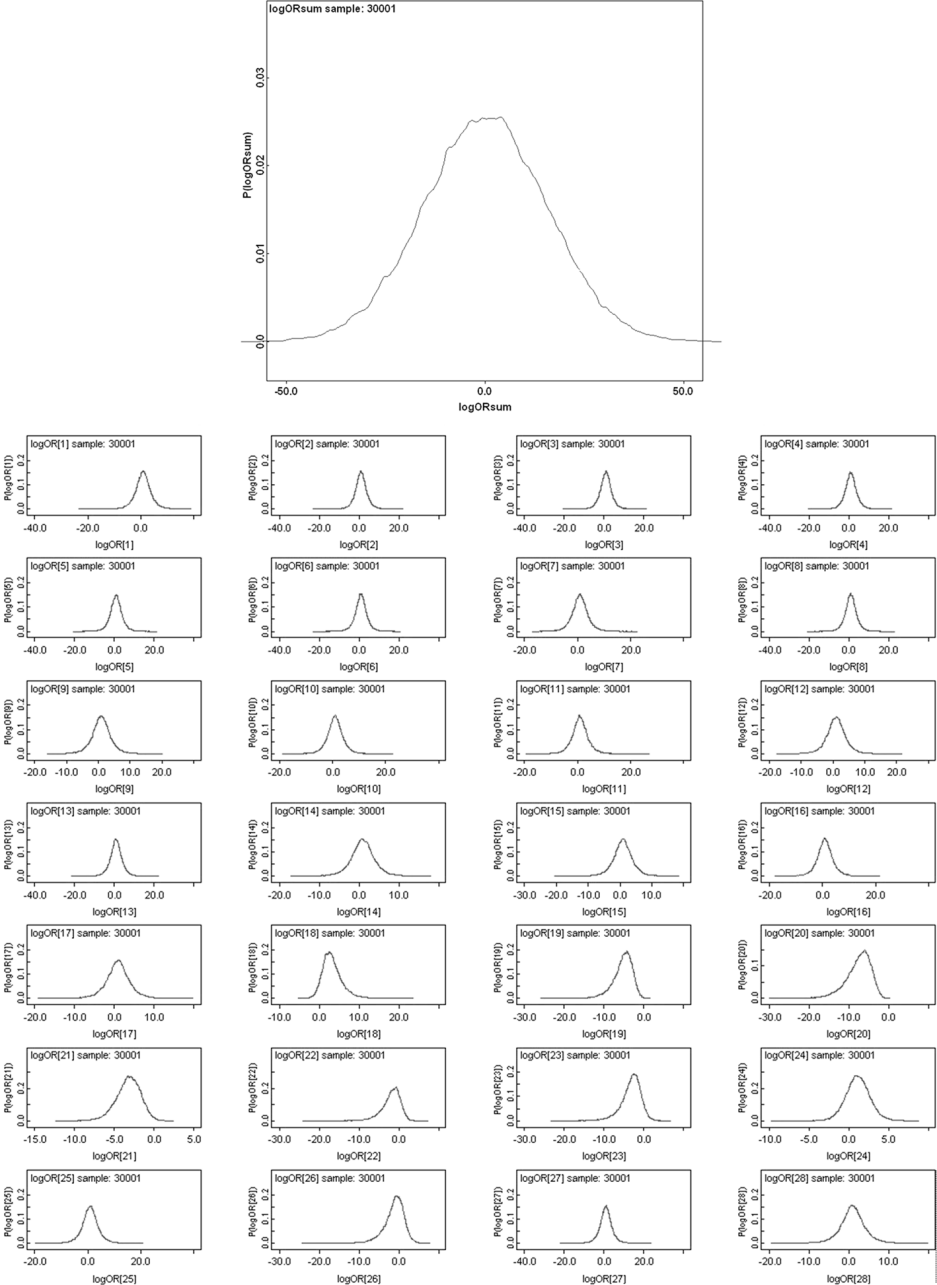
Posterior distribution of log (odds ratio) for the comparison of the observed Malpighian tubule guanine associated with anterior versus posterior gut regions (not including the rectal vesicle) in *Pergamasus longicornis*. *Upper* Over all time points. *Lower* For each distinct time point separately (here indexed as logOR[1....28] where first row is for distinct time points [1] [2] [3] [4], second row is for time points [5] [6] [7] [8] etc). Note: skew distributions for logOR[18] = 2 days after start of feeding, logOR[23] = 7 days, logOR[26] = 10 days etc)

The Bayesian posterior distribution of the log odds ratio derived for the estimated probabilities at each time point together with that for the overall occurrence of guanine in Malpighian tubules associated with anterior gut regions versus posterior gut regions is shown in Fig. 5.

**Fig. 6:**
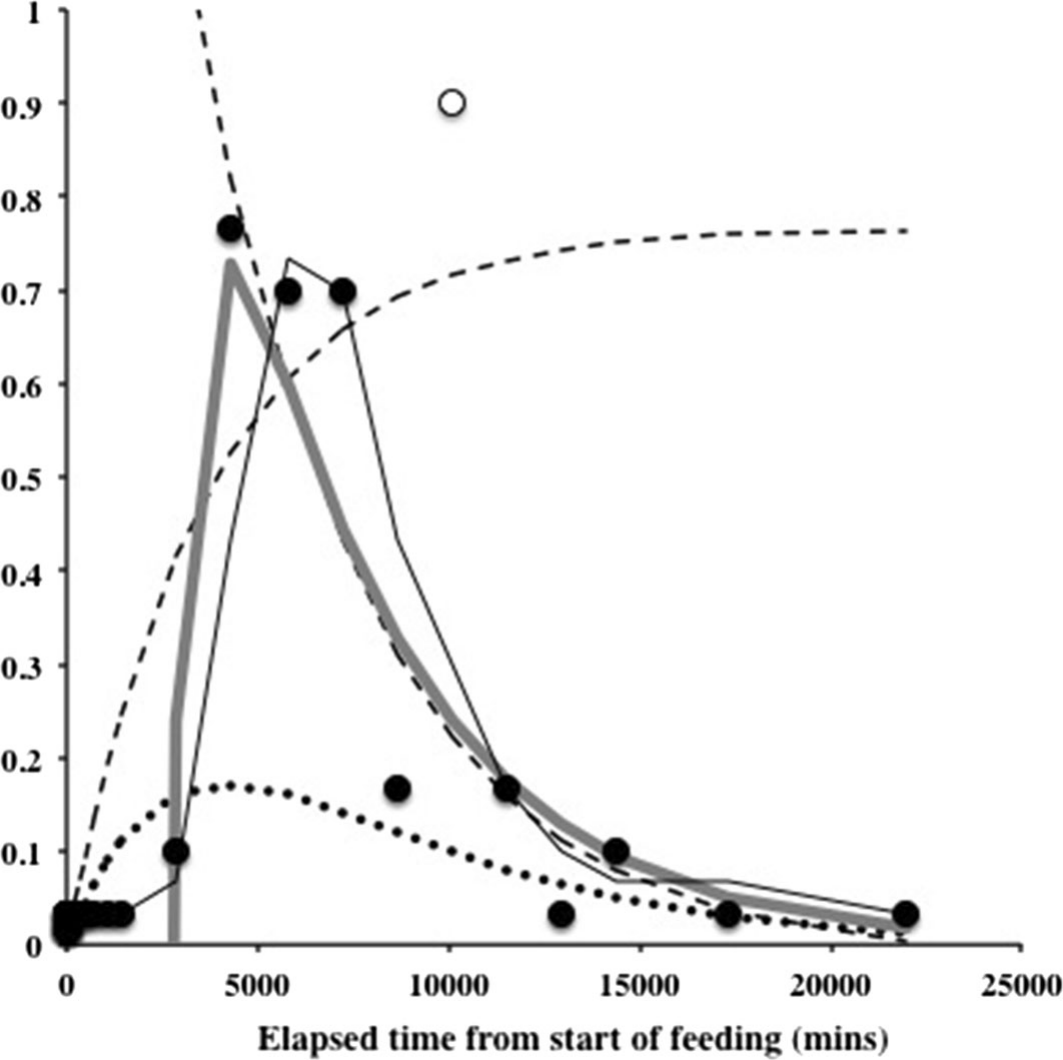
Kinetic modelling of the Bayesian posterior mean probability of Malpighian guanine occurrence (over all associated gut regions—not including the rectal vesicle) in *Pergamasus longicornis*. *Black filled circles* (and *white open circle* outlier at *t* = 7 days) are Bayesian posterior mean estimates. *Solid thin black line* two point moving average smoother over fitted probability (omitting outlier). *Descending dashed thin black line* estimated first order elimination from the tubule system (omitting outlier). *Rising dashed line* indicative profile of simultaneously estimated first order input ($t_{\frac{1}{2}}=1.8\ days$) into the tubular system (omitting outlier and linearly rescaled so asymptote is $M[t_{max}]$ for ease of comparative illustration—real curve higher off the scale). *Line with small crosses* actual sum of the two exponential model (i.e. simultaneously fitting $M[t] = elimination-input$)] omitting outlier). *Solid grey line* Malpighian tubule guanine occurrence model of *elimination* less *multiplicative similar inputs*, now with effective input of $t_{\frac{1}{2}}\equiv 7.8\,h$—see text)

Results of the kinetic modelling of the Bayesian posterior mean estimate of the probability of guanine in the Malpighian tubules are shown in Fig. 6 on an arithmetic time scale. The exponential models would not converge satisfactorily without omitting the outlier at 8 days. The large time difference between the appearance of Malpighian guanine (48 h—10 days post commencement of feeding) and the swop to gut emptying-predominating at 2 h together with the median commencement of gut emptying at 12.5 h (see Bowman 2014) confirms that assuming assimilation has reached its asymptotes as in the Materials and Methods (above) is an acceptable simplification.

## Discussion

No reports of mites being ammonotelic or ureotelic could be found. As is the case in many other mites (e.g. Calyptostomids—Vistorin-Theis 1977), a pair of Malpighian tubules lie in the haemocoel of *Pergamasus longicornis* one on each side of the animal along the whole length of the mite closely associated with the various gut regions (see Fig. 2). Closed at one end, they debouch ventrally into the rectal vesicle in this mite (like in tyroglyphids—Oboussier 1939, and as in the ticks *Rhipicephalus* and *Ixodes hexagonus*—Blanc 1910). In general, Malpighian tubules are thought to play an excretory function rather than an osmoregulatory one (Fretter and Graham 1976), yet appear to be absent in glychyphagids (Oboussier 1939). In this histological study, they were usually contracted and empty of excretory products—their ends being not noticeably enlarged. As in some ticks (see Oliveira et al. 2012) but not others (see Balashov and Raikhel 1976; Shoura 1988), cells appeared to be of a uniform gross structure. As in ticks (see Balashov and Raikhel 1975) there was also no distinct division of the Malpighian tubules into departments.

Although no obvious advantage of guanine excretion over xanthine excretion is apparent in purinotelic invertebrates, the excretory crystals in *P. longicornis* are taken to be guanine rather than xanthine or uric acid on several counts. Unlike *Palamnaeus bengalensis* (see Kanungo et al. 1962), *P. longicornis* does not appear to be uricotelic. In its pure crystaline form, uric acid is white but in biological samples produces finer, more stellate, needle like crystals out of solution or spherical gravel-like to millet grain-size uroliths in excess (see Furman et al. 2015). Neither match the crystals seen visually in this mite study. Xanthine in its pure form is also white and crystaline but produces rounder, darker coloured amorphous crystals in xanthinuria samples. Again not matching that seen in the *P. longicornis* system. Rather, the Malpighian tubule crystals seen were squarish/rhomboidal (like in *Latrodectus pallidus*—see e.g. Levy-Lior et al. 2010), brilliant white/silvery and ‘flaring’ in behaviour under light. For visible light, most transparent media have refractive indices between 1 and 2. Crystals with a high index of refraction partially reflect and transmit light from layer to layer thus producing a pearly lustre as observed in the pergamasid. The refractive index for uric acid, xanthine and guanine is: 1.721, 1.989 and 2.047, respectively (see www.chembk.com). Refraction causes dispersion and birefringence—the greater the index the greater the effect. Unstained biological structures appear mostly transparent under bright-field microscopy, not attentuating appreciable quantities of light. Here in the Malpighian tubule crystals of *P. longicornis* birefringence and partial reflectance was seen like in the matt-white colouration (Levy-Lior et al. 2010) and reflection structures (see e.g. Jordan et al. 2012) made from guanine in other animals. One cannot be certain—specific confirmatory tests with guanase awaits further work—but for this histological paper the large, white, glistening Malpighian excretory crystals observed are taken to be guanine.

To answer the various questions posed above in the introduction to this study:

### Whether the state of expansion/contraction in Malpighian tubules is different anteriorly from that posteriorly (as is the case for the gut—see Bowman 2014)?

The symmetric overall log(odds ratio) posterior distribution (Fig. 5 *Upper*) indicates not. It shows no clear evidence of distinction in guanine occurrence overall between the Malpighian tubules associated with anterior gut regions versus those associated with posterior gut regions in this small sample (mean = 0.1239 sd = 15.73). The Malpighian tubule system appears to behave in general as a single uniform process. However, between 2 to 7 days (and perhaps through to 10 days) after the start of feeding there is some mild evidence of a distinction—for the most part the anteriorly gut-associated tubules have a lower occurrence of guanine than the posteriorly gut-associated ones (Fig. 5 *Lower*). Unlike the gut expansion/contraction itself (Bowman 2014), Fig. 3 shows that there is no clear evidence for a LIFO (last-in first-out) or a FIFO (first-in first-out) process in the Malpighian tubules.

### Is there evidence of Malpighian expansion with fluid during the ‘concentrative’ phase of pergamasid feeding (i.e. 10–90 min from start of feeding)?

Unlike the gut, no expanded sections of Malpighian tubules were observed to be empty of crystal contents—showing that never just fluid was collected in them. There is no evidence that the Malpighian tubules play an obvious role in fluid balance during feeding in *P. longicornis*. In particular, Fig. 3 shows that there was no evidence of Malpighian expansion *early on* in feeding as a putative fluid balancing mechanism to concentrate ingested prey material in the gut (see Bowman 2014). Coxal droplets still appear to be the simplest homeostatic explanation.

### Is crystalline guanine present during the early ‘anal dabbing’ phase (i.e. <1.5 h after start of feeding)?

**Fig. 7:**
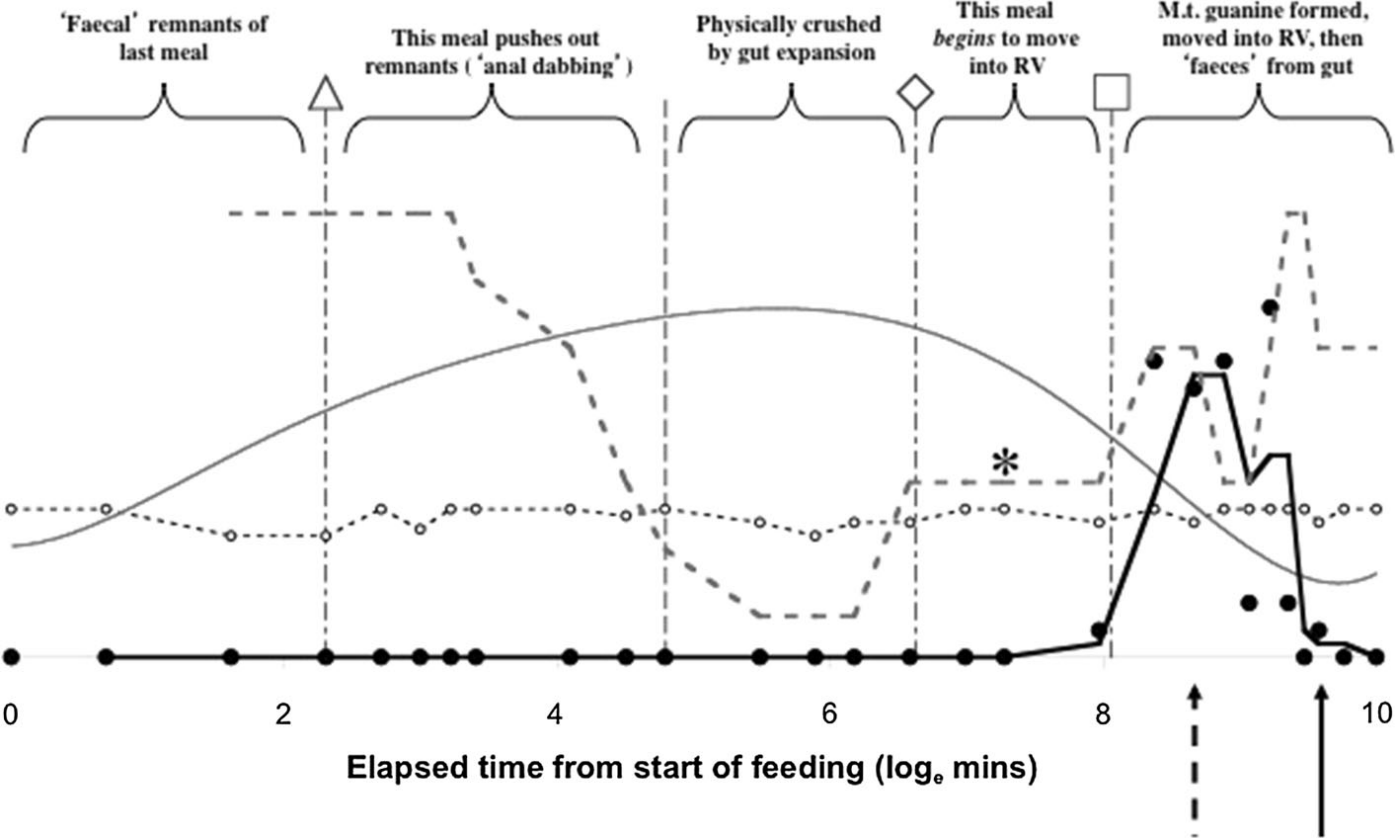
Re-scaled schematic of observed guanine crystal presence during feeding and digestion in *Pergamasus longicornis*—observed data. Annotation at top is proposed physiological interpretation and effect upon the rectal vesicle conditional on no idiosomal expansion. *Small circles and dotted data line* = Lumenal guanine mean score in rectal vesicle (showing continual presence of guanine crystals in rectal vesicle). *Large closed circles* and heavy solid two point moving average smooth trend = Overall Malpighian tubule guanine mean score (showing late peak plateau about 4 days after the start of feeding marked by *Dashed vertical arrow*). *Grey dotted* third order moving average smooth trend = Rectal vesicle expansion/contraction mean score (see data in Bowman 2014) showing it is $180^{o}$ out of phase with the overall gut (less rectal vesicle) expansion/contraction mean score—*grey solid* sixth order polynomial smooth trend. Time is from the commencement of feeding and is on a natural logarithmic scale. *Grey dashed line* centrally (with no accompanying symbol) is at the 120 min breakpoint between ingestion dominating and digestion predominating (see Bowman 2014). Y axis is a measure of average size and infers relative amounts between plotted points within a line but not absolute values between lines. *Square* indicates worse-case total feeding cycle time based upon Bowman (2014) modelling gut expansion/contraction (=52.5 h), thereafter is excretion. *Triangle* indicates best estimate of initial completion of gut filling time (10 min—see Bowman 2014). *Diamond* indicates best estimate of time of commencement of initial gut emptying (12.5 h—see Bowman 2014). Rectal vesicle expansion around 18 h (log$_{e}$ time = 7) after the start of feeding marked by *asteristic* due to latent egestive fraction. *Solid arrow* indicates a conservative suggestion for the beginning point of hunger/starvation (10 days)–note the disappearance of Malpighian tubule guanine and how the rectal vesicle moves back to an initial pre-fed state thereafter

Figure 3 indicates definitely not for the Malpighian tubules, but in contrast certainly this is true for the rectal vesicle (see Fig. 4). Moreover, not even in the most posterior parts of the Malpighian system (i.e. those associated with the rectal vesicle itself (see Fig. 3) was there any evidence of expansion during the ‘anal dabbing’ phase—pointing to the deposited fluid and crystal material described in Bowman (1984) being from the rectal vesicle itself. This anal dabbing appears to be driven by the increasing crushing of the rectal vesicle as the gut fills up over time (see Figs. 7, 9). Physicality within a constraining all enveloping ‘suit of armour’ appears to drive this relationship.

### Is the time course of guanine production congruent with a slow digestion phase predominating after 120 min from the start of feeding?

Again—no. Guanine crystals were only found in expanded Malpighian tubules during the period 48 h to 10 days after the commencement of feeding (see Fig. 3). This is after the gut has been emptied (at 52.5 h—see Bowman 2014). Crystals were not produced anywhere during the slow phase of digestion after the physiological ‘knot point’ of gut filling-predominating versus gut emptying-predominating. This suggests catabolic guanine production is well downstream of the digestion of a discrete pulse of food. A hysteretic relationship is present (see Fig. 9) between the gut size and guanine occurrence in the Malpighian tubule system. Whatever the pathway is in detail between the gut and Malpighian excretion—attenuation and delay occurs. Figure 10 proposes, at least, passage through a haemocoel compartment if not also through multiple catabolic processes as the cause (see further discussion sections below).

### Are excretory products only produced in the later phase after the mite has stopped feeding?

Yes—excretory products are only produced in the Malpighian tubule system late on post-prandially—well after the mite has stopped feeding (and presumably digested its meal). However, from Fig. 4, apparently gently randomly fluctuating levels of guanine are permanently present in the lumen of the rectal vesicle. This supports Bowman (2014)’s suggestion that metabolism should produce excretory products throughout the digestive period. Yet, the pattern of this excretory ‘background’ bears however no resemblance to any of the detailed patterns in other observed phenomena (i.e. the gut size changes seen by Bowman (2014) nor the Malpighian guanine presence in this histological study herein). Despite the gut size changing over time, not all of the contents of the rectal vesicle can therefore be mechanically voided by the physical mechanism of overall changes in internal idiosomal volume occurring during prey ingestion. Interestingly, there is not a huge rise in rectal vesicle guanine contents late on. The episodic guanine in the Malpighian tubules is not reflected by an episodic occurrence of guanine in the rectal vesicle. There is no unequivocal evidence for the storage in the rectal vesicle of a discrete ‘pulse’ of Malpighian excretory products from a discrete ‘pulse’ of prey ingestion—whether arising from the current meal or the previous meal.

Where does the Malpighian tubule guanine go? Rectal vesicle guanine must be being cleared when accumulated. It cannot be that the Malpighian tubules do not discharge at all into the rectal vesicle as post-prandially they themselves *are* devoid of guanine. Perhaps the rectal vesicle behaves like an overflow regulator—losing guanine as fast as it gains it from the Malpighian tubules. It is tempting to conclude that the early occurrence of guanine in the rectal vesicle probably arises from catabolism of the *previous* meal, and the later occurrence of rectal vesicle guanine crystals probably arises from the degradation of the *current* meal. In that way, a pergamasid mite constrained from marked expansion by its rigid exoskeleton would be able to feed again before the handling of the previous prey is completely finished (see Lister et al. 1988).

One could, of course instead, interpret this continual level of guanine in the lumen of the rectal vesicle as coming from a different non-episodic non-Malpighian source–perhaps directly via the hind-gut from digestion and catabolism in situ within the gut itself. The latter hypothesis is testable. If it is true, there should be evidence of ‘excretory production’ somehow by the gut itself (whether intracellularly in the gut wall, or extracellularly in the gut lumen) on detailed histological inspection. However, as large crystalline guanine was not found in substantial quantities anywhere else in the gut in this study, this production would have to manifest itself by levels of an excretory product leading to guanine, rather than guanine crystals themselves. This intermediate might be found in the lumen of the gut sometime during prey digestion (even if intracellularly produced). Moreover, such a proposed product should be absent early on during feeding, then increase during digestion, before decreasing as this pre-guanine material is moved to the rectal vesicle. Considering the low scale of crystalline material continually present in the rectal vesicle, these gut produced amounts of ‘waste-stuff’ would only need to be fairly low with a shallow time course of increase during digestion. Could they be easily missed? Could they be sequestered in the gut cells or mixed in with the ingested prey material in the gut lumen? Careful intensive examination of the lumenal contents of the gut in this predatory mite during digestion for such latent material needs to be made in follow-up work. A potential ‘missing fraction’ seems to be indicated. This fraction would cover the physiological period of egestion (see Fig. 10).

The overall feeding cycle finishes at 52.5 h. By this time, gut egestion must have commenced—marked first by any such gut waste-stuff (if produced) moving into the rectal vesicle (see first bump in Fig. 7); then followed by excretion proper as there is a rise of Malpighian tubule guanine crystals, which are finally voided into the rectal vesicle (for disposal too). This tubular emptying is by about 9.5 days after feeding—when fasting may commence in *P. longicornis*. This nicely matches the unsubstantiated claim by Bowman (1984) that the length of ‘digestion’ was approximately one week and better matches estimates of the biological half-life of ingested material in cryptozoans ranging from 5.5–27 days (Reichle and Crossley 1965). Careful follow-up examination again of the lumen of the gut and rectal vesicle for membranous or faecal material could also help clarify matters here.

### Does the time course of guanine production match the estimated total digestion time?

Previous sections above discuss the topics of Malpighian tubule guanine during the early ingestive and concentrative feeding phases together also with the early and slow digestion phases. However, what is happening with excretion during the intervening period i.e. between the estimate of Bowman (2014) of 52.5 h for complete feeding and digestion, and the observed late pulse of excretion around 4 days after the start of feeding—Fig. 7? Also, what is happening beyond this period?

There is one clear possible explanation for the occurrence of Malpighian tubular guanine not matching the period between slow digestion and 52.5 h—Bowman (2014) was incorrect and produced an underestimate when he derived the total ingestion and digestion time for *P. longicornis* feeding on larval dipteran prey. However, inspection of his figures (cf. 97.5% centile plus 2 typical errors for hind gut emptying => 25 h from commencement of feeding) shows that even under the worse case scenarios his data on the expansion/contraction status of the gut in *P. longicornis* does not support digestion taking up to 10 days! This suggests that measuring gut expansion/contraction is not a good surrogate of *all* aspects of the digestive physiological status of this predatory mite. It may measure well the rapid imbibition during, and the slowly changing digestive status, after prey feeding; but does not well measure consequent assimilation and the metabolic production of ensuing excretory material after full and final digestion. Gut physicality does not inform all of physiology.

So, how can one explain the disappearance of material from the gut indicated by gut contraction by 52.5 h (after feeding commences) *without* the coincident appearance of excretory breakdown products in the Malpighian tubules? If there was a long series (of histologically unseen) catabolic changes from the primary prey ingested material expanding the gut—through assimilation of material off into the haemocoel and other notional compartments—to the appearance of final guanine in the expanded Malpighian tubules; there would be just this hysteretic effect seen herein. Peak gut size changes would precede peak Malpighian tubule guanine levels (see Figs. 7, 9). Hysteresis is a common phenomenon in biology—just because two phenomena are not exactly coincident in time does not preclude a causative link between them. A plausible mechanism is just needed (see Bradford-Hill 1965). All that is necessary here is that the prey material becomes digested and assimilated before being catabolised to excretory products. How could this be operationally detected without explictly observing haemocoel changes?

Haemocoel changes depend upon an appropriate input ‘echo’ from the previous compartment before they change. Given the linked integrative nature of an animals physiology (see Fig. 10), finding evidence of a latent catabolic ‘egestive’ fraction in the data as a surrogate mechanism congruent with the delay between the two peaks in the results of this histological study is a sufficient clue. This can be achieved simply by posing a *common* degradative process for foodstuff and an assumption that the haemocel is a very well stirred homogenous compartment with essentially instant equilibrium kinetics. The former conjecture (marked as grey arrows in Fig. 10) is a prefered simpler assumption than assuming egest and faecal production from prey food is fundamentally different than the guanine-forming catabolism of prey food (Occam’s razor applies !). This could be explicitly tested by examining estimates for *C* versus those of *E* in future work. The latter conjecture of panmixis seems likely given the open nature of the haemocoel bathing all organs in pergamasids and the prescence of a pumping heart dorsally. Figure 7 indicates indirect evidence for just this partly assimilated—partly catabolised ‘echo’ marked by the rise labeled with *. This should be explicitly searched for in follow-up work of changes to gut lumen contents and be checked as to its adequacy in mirroring haemocoelic catabolism (i.e. if the rate constant *E* is of the order of *C*?).

There is some oblique evidence for such a possible catabolic signal of a surrogate ‘egestive fraction’ in previous work on this mite (Bowman 1985, 1987). Here high protein content values (postulated to indicate nutritive status) were positively correlated with visually observed dark material inside the idiosoma (especially in the larger female *P. longicornis*). Bowman (1985) interpreted this dark colouration as prey material inside the gut indicative of the mite having fed. Thus high (dark) food protein = high protein content he observed. However he did not describe any white or refractive material visible through the cuticle of any of the 100 or so mites he examined—which one would expect from the guanine undoubtedly present in some of them. If the dark colour was in fact a guanine ‘shadow’ then protein levels should be low assuming full meal digestion by then given the results herein of this histological study. That is if the mite is dark = guanine, then protein content should be low (it all having been catabolised to carbohydrate/fat and its nitrogen atoms having being turned into guanine). That is inconsistent with his 1985 and 1987 results. So rather could in fact a part of this observed dark colouration also be a latent egestive fraction itself deeper inside the idiosoma hidden by the thick integument? That is, there is still some pre-guanine/partly catabolised proteinaceous prey material in the gut when a mite is scored as ‘dark’? This is not as fanciful as it first seems.

Bowman (1987)’s statistical model based upon idiosomal colour only explained 28% of the variation in protein content, which points to other important non-visible determinants of nutritive status or a strong degree of confounding in the origin of the dark colouration. Could in fact the results of Bowman (1985) be explained better by the protein assay picking up the pulse of ‘invisible’ partly assimilated—partly catabolised prey material after digestion (an ‘egestive fraction’)? Then the darkness of the idiosoma is a gut/haemocoelic surrogate for the pre-guanine crystal content being produced deep under the opaque idiosomal integument (rather than just evidence of ingested pigmented prey)? Since the two peaks of gut expansion on feeding and that of Malpighian guanine appearance shown in this study herein do not show exact temporal coincidence (only agreement with an effective delay), poor predictability (just like Bowman 1985’s results) of one from the other (without a lag term) would be found. This idea that the ‘egestive fraction’ may be mid-gut-borne pre-excretory catabolic products shadowing haemocoelic changes preceding the later guanine crystal formation inside the rectal vesicle can be confirmed or refuted if micro-histological spectrophotometric or biochemical assays for guanine and its precursors are followed-up in a new collection series of mites.

### Are guanine crystals mechanically voided from the body by the physical mechanism of overall gut size changes?

Turning first to the rectal vesicle. One can see from Fig. 7 that the size changes in the rectal vesicle are approximately $180^{\circ }$ out of phase with those of the rest of the gut. The rectal vesicle appears to be a passive storage vessel emptied in part by the expansion of the mid- and hind-gut with ingested food (see Fig. 9). Peak rectal vesicle contraction is around the 5 h mark after the commencement of feeding. Peak expansion in the rest of the gut overall is perhaps marginally earlier of this time. Although in an opposite direction to each other, there is no evidence for a lack of synchronicity between the two—they inversely change size in step. The legend at the top of Fig. 7, offers a schema for the physical origin of these rectal vesicle size changes.

Turning now to the Malpighian tubular system. Certainly the Malpighian tubules expand with increasing guanine contents during the period when the gut is contracting and the rectal vesicle is enlarging (see Fig. 9)—a physical exchange of idiosomal volume thus between them appears likely. However, is there evidence that the guanine in the Malpighian tubules is mechanically voided by the physical mechanism of overall gut size changes within the idiosoma ? Apparently not—Fig. 9 (marbled arrows) shows the Malpighian tubules empty into the rectal vesicle over the time when the gut is still contracting ! Yet the rectal vesicle never shows a large depot of guanine (see Fig. 4). So how is Malpighian tubule material lost from the tubules? Bowman (1984) describes the occasional deposition of small clear droplets (with granular material) from the anus in the later phase of feeding but not wholesale deposition of guanine or faecal material, or how this expulsion might occur. The histology in this study herein can shed no light on this either. So, how the Malpighian tubules are emptied (*and they are*—see Fig. 3); and how the rectal vesicle itself is emptied (*and it is*—see Bowman 2014) of any pulse of Malpighian tubule material or of gut materials (*at other times than when it is crushed by gut filling*); remains a mystery. Could an all enveloping smooth muscle layer over the tubules and the rectal vesicle be the contractile cause? Coons and Axtell (1971) describes the excretory tubes of *Macrocheles muscaedomesticae* (Mesostigmata: Macrochelidae) as being partially ensheathed in fat-body and invested throughout by a branching system of visceral muscles. Does this periodically ensure the voiding of any pulse of Malpighian tubular material arriving—like an overflow regulator? Is this what is happening during ‘anal dabbing’? Is it even right to assume by default that the debouchment of rectal vesicle contents is in fact in common with that of Malpighian guanine voiding? That is, are faeces deposited separately from Malpighian guanine? Could Malpighian tubule musculature be independent of that of the rectal vesicle? Could the physical design of the pergamasid pygidium play a defining role in this as in argasids (Roshdy and Hoogstraal 1974)? Further timed observations of mite behaviour associated with defecation; measurement on the substrate of what and how much material is deposited; together with detailed ultrastructural investigations of the mite’s Malpighian tubule and pygidial musculature is needed.

### What can be said of any coupling to gut changes using simple kinetic models?

The peak estimated probability of guanine in the Malpighian system is around 3–5 days after feeding starts (Fig. 6). The elimination of guanine from the Malpighian system is slow ($t_{\frac{1}{2}}=53$ h). This magnitude matches the estimate for the overall feeding and digestion (and egestion) cycle time of 52.5 h (Bowman 2014) indicating that there is very very slow loss of excretory waste products in comparison. From its estimated peak probability, using the common rule that 5 half-lives equates to a 97% drop (Hacker et al. 2009), this would suggest that the guanine pulse from a single dipteran larval meal is totally eliminated from the Malpighian system by about 15 days after the start of feeding. As the observations show (Fig. 3) pre-feeding low levels are seen by 12 days after feeding commences—this is equivalent to approximately 4 half-lives or a 94% decline. This is probably after hunger/starvation sets in (at 10 days?). Input into the Malpighian system as measured by guanine appearance asymptotes around 9–10 days (again pointing to hunger/starvation commencing then perhaps?). The estimated input half life is shorter (about 43 h) than that of output with an estimated 5 half-lives of almost 9 days. This too broadly matches the suggested observed beginning point of hunger/starvation in Fig. 7. Complete catabolism of a single prey meal thus takes a very long time compared to feeding (i.e. compared to 56–96 min—a factor of 166–285 times longer!). The mite’s gut and excretion is truly a stiff system.

However, as can also be seen in Fig. 6, a simple sum of two exponentials derived from standard simultaneous fitting by a ‘stripping’ process poorly approximates the smoothed curve of actual observations. Malpighian tubule guanine occurrence appears not just to be an attenuative ‘blurring’ of the original 58–96 min of prey input. Little seems to happen until around the time of the commencement of initial gut emptying (12.5 h—see Bowman 2014) when a power law increase appears through to about 5 days . How to allow for this? Adding similar sized input terms as if ‘multiple dosing’ of ingested food was occurring simultaneously i.e. augmenting $a_{in}$ to say $n\cdot a_{in}$ relative to $a_{el}$, does not improve matters (*results not shown*). This would be like the mite eating a fly larva and then another fly larva again and again and again. That was not the case experimentally in this study. Serially adding such ingestion pulses at delayed intervals or subject to partitional delays (through equilibrium with notional effect compartments) would similarly attenuate the extent but not solve the observed issue of a sharp peak. That is, eating a larva then waiting and eating another larva then waiting and eating another larva etc in fairly quick succession is not the answer. The basic input model is insufficient on its own to explain the observed data.

On the face of it, excretion does not appear to be straightforwardly linked to ingestion and digestion. Relative to Malpighian tubule guanine elimination, much larger *and* much steeper input processes are needed. Deattenuation of the ingestive pulse is needed to match the observed Malpighian tubule profile of guanine occurrence. One option is that in fact no guanine loss is occurring during its accumulation, and then elimination is triggered at an arbitrary time point (here 3 days after the commencement of feeding). Discontinuously, fitting this guanine input on its own as an episodic process suggests a $t_{\frac{1}{2}}=10.5-16.7$ h depending upon where the accumulation is first fitted from. However, posing a special ‘delay’ before physiological processes kick-in is not actually necessary. Instead, if one rather assumes that the Malpighian guanine system receives an interacting cascade of *multiplicative* inputs of the same fundamental nature (equivalent heuristically to the fitted exponential input contribution being raised to a power—here denoted *n*) then a much better fit is obtained to the observations (see thick grey line in Fig. 6). A ‘delay’ appears naturally (simply by sacrificing the knot condition). Moreover the intercept with the horizontal abscissa now matches well the worst-case scenario for total feeding cycle time derived from gut expansion-contraction data of 52.5 h (Bowman 2014). Effectively what this is saying is that the food ingestion pulse as it passes through the various gut and metabolic systems on the way to resulting in Malpighian tubule guanine, does not become spread out, diminished and linearly summed by passage from notional physiological compartment to compartment. Rather it is stoichometrically augmented and amplified in overall rate and extent by the repeated application of, and synergistic interactions between, the *same* kernel generative ‘absorption’ process. The consequences of a simple input pulse of larval food is amplified and synergistically sharpened. It is not that this is an *n*th order chemical reaction—which is metabolically rare and unlikely to occur—this parameter (n) is used simply empirically in the model to infer what might be going on.

Now, the fitted equation can just be taken to be

$$\begin{aligned} M[t]=\hat{a}_{el}.e^{\hat{b}_{el}.t}-\left[ \hat{a}_{in}.e^{\hat{b}_{in}.t}\right] ^n \end{aligned}$$

where the estimates (of *a* and *b*) were from simple exponential stripping. A power of $n=5.5$ was found empirically suitable. This power law trick effectively changes the extent $a_{in}$ to $(a_{in})^n$ (thus sacrificing the smooth knot between the two processes) and shortens the input half-life by a factor of $\frac{1}{n}$ through making $b_{in}\rightarrow n\cdot b_{in}$ i.e. it becomes more extreme (effectively $t_{\frac{1}{2}}\equiv 7.8$ h). However, no claim that a power law mechanism is the actual biology involved is being made as many functional forms can result in such a log/log relationship. The powering term is simply a surrogate summary over a set of interacting input processes of the *same basis* to yield a time varying rate and extent. Another way to look at this artifice is that ensures more input from the previous notional compartment arriving early on (to become Malpighian tubule guanine) and less arriving late on when the input is naturally fading away. If the input was thought of as an infusion pump, then the gain on the pump is turned up much higher than expected at first and then turned progressively down over time (this would be poorly modelled by simple signal ‘stripping’). Proteretic de-blurring of a signal or the ‘piling up of the initial strength’ in this way of a signal is evidence of a either physiological cascade or an accumulation and episodic release system (like an automatic flushing toilet). However, no evidence of repeated episodic release is present. Rather the data supports simple elimination at a constant rate over a long time period. Not only that, but it is biochemically reasonable that Malpighian tubule guanine production with respect to prey input might result from a physiological cascade—each process reinforcing the other. In other words, a rapid run-away system of catabolism inputing into the Malpighian tubule system is not implausible. Looking for mutually connected and dependent gut cellular processes all leading to guanine production in follow-up work would help substantiate this idea.

**Fig. 8:**
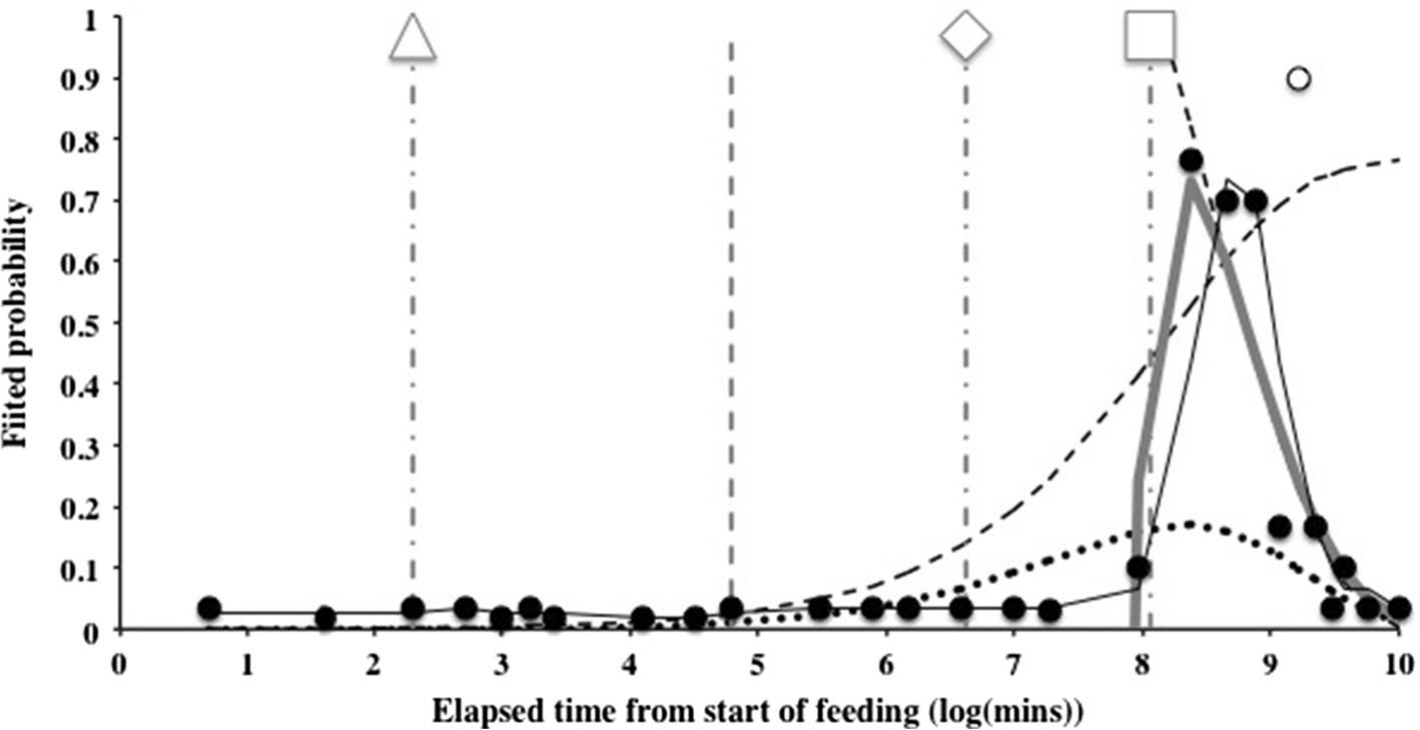
Back transformation of kinetic models of Bayesian probability of guanine occurring in the Malpighian tubules over all gut regions (not including the rectal vesicle) in *Pergamasus longicornis* onto linear observed scale. Line types and data symbols as in Fig. 6. *Square* indicates worse-case total feeding cycle time based upon Bowman (2014) modelling gut expansion/contraction (52.5 h), thereafter is egestion. *Triangle* indicates best estimate of initial completion of gut filling time (10 min—see Bowman 2014). *Diamond* indicates best estimate of time of commencement of initial gut emptying (12.5 h—see Bowman 2014). Note a *basis* input into the Malpighian tubule excretory system effectively needs to begin during the proposed egestive gut phase as the rectal vesicle itself also begins to expand (see Fig. 7)

Figure 8 summarises the kinetic analysis on a $\hbox {log}_e$ time scale. This indicates that the basic fitted input process and its contribution (without powering) would need, as a corollary, a latent rise in the *basis* determinants of Malpighian guanine to exist from about 4–8 h after the commencement of feeding. This point is before the best estimate time of initial gut emptying, but after the digestive switching knot point at 2 h. The latent fraction would then continue to increase through 18 h and up to the regular appearance of guanine in the Malpighian tubule system at 48 h. This is just the phase of the proposed ‘hidden’ egestive fraction that drives rectal vesicle swelling over this time (* in Fig. 7). The physiological cascade then sharpening this fundamental response to a discrete input pulse of prey arriving into the excretory system. It is very tempting to conclude that the egestive fraction is a part of the precursor physiology to Malpighian tubule guanine occurrence (or at least in close synchrony with it through common processes). Examination of gut lumenal contents and any haemocoel or changes over this time slot and their rates could be very informative in addressing this. Perhaps it might be possible to find evidence for gut cell degeneration releasing egestive material (like the residual bodies in ticks—see Raikhel 1978)?

### Does the excretory system too exhibit “stiff” behaviour?

**Fig. 9:**
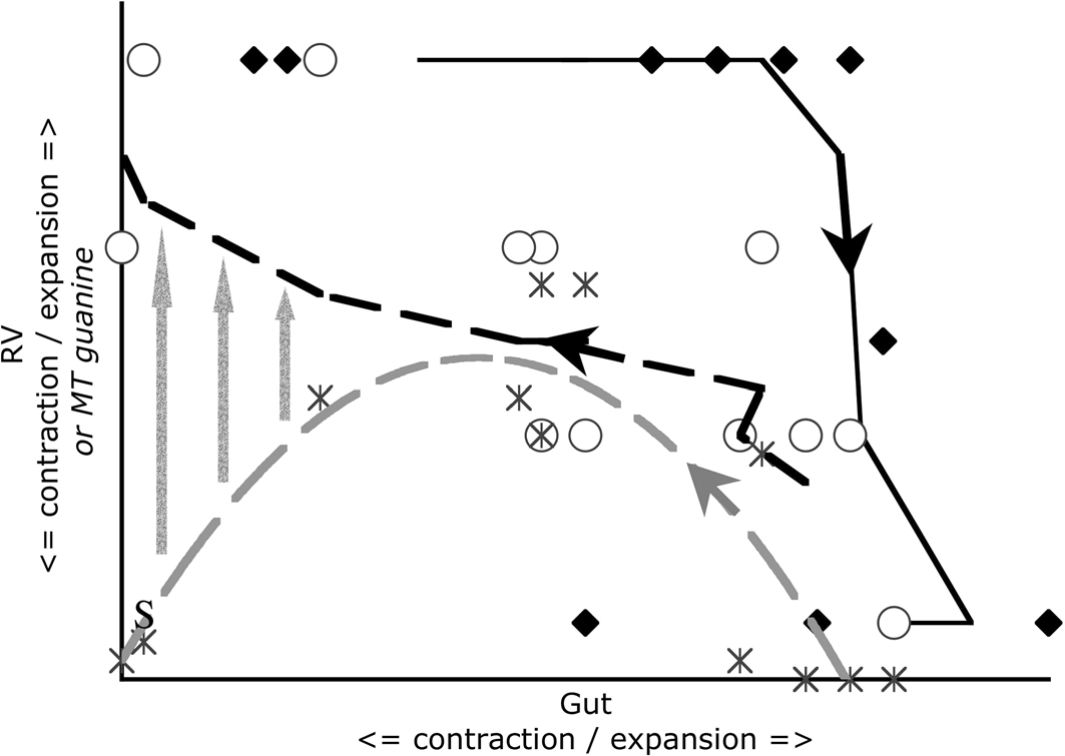
Time ordered plot showing hysteretic relationship of Malpighian tubule status in *Pergamasus longicornis*—observed data from this study, and gut status from Bowman (2014). *Solid line with black arrow and black solid diamonds* show that as the gut expands the rectal vesicle is squashed (0 min to 6 h after start of feeding; 3rd order moving average trend). *Dashed line with black arrow and grey open circles* shows that as the gut contracts the rectal vesicle expands (8 h to 10 days after start of feeding; 4th order moving average trend). *Dashed grey line with grey arrow and grey stars* shows initial Malpighian tubule expansion with guanine crystal accumulation as the gut contracts (8 h to 10 days after start of feeding; quadratic trend) followed by tubular contraction/emptying as guanine is transferred to the rectal vesicle by an unknown mechanism (*grey marbled vertical arrows*, 8–10 days after start of feeding). Fasting/starvation (marked by ‘S’) starts around 9 days from the start of feeding

**Fig. 10:**
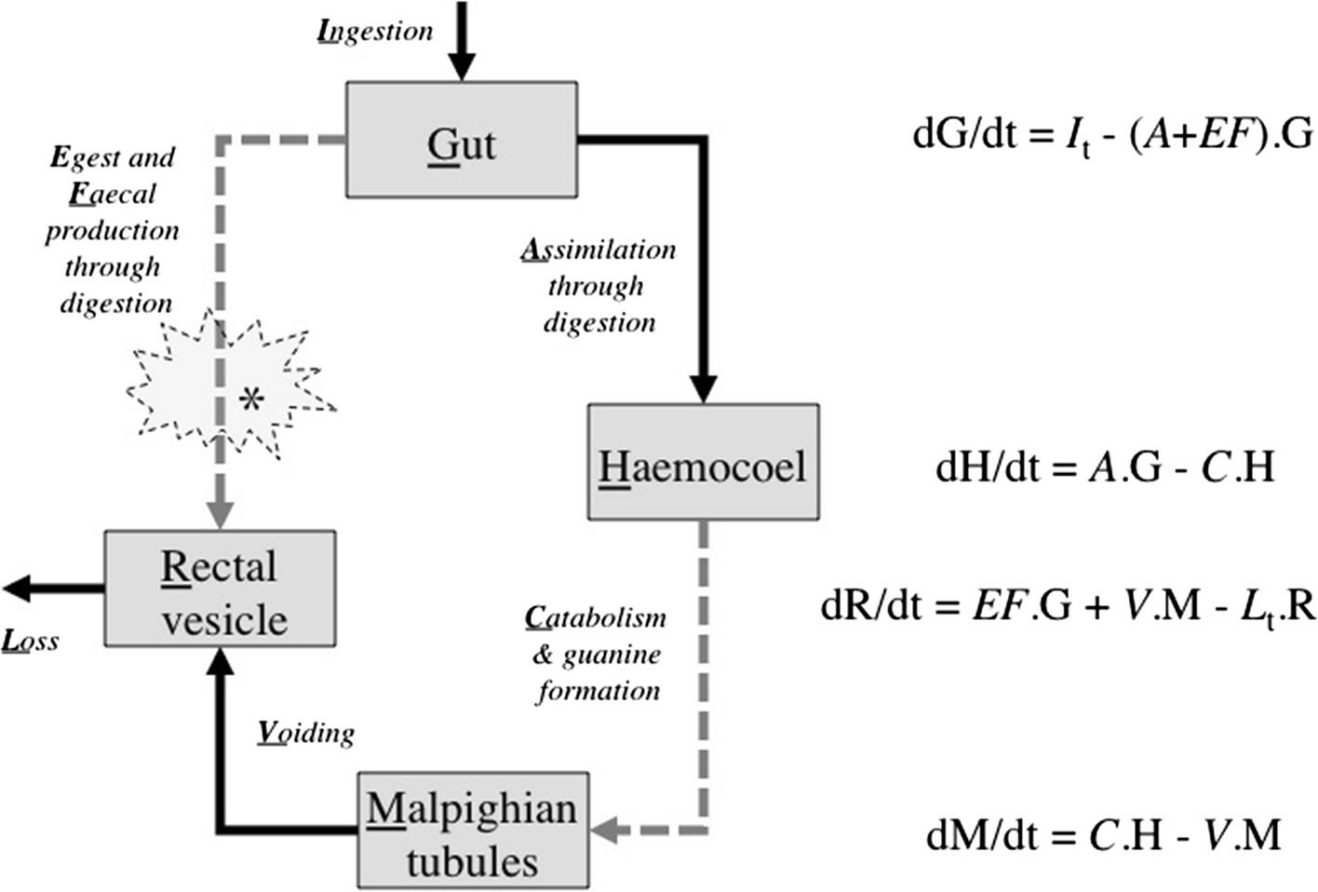
Summary scheme and overall simple first-order linear differential equation basis for decomposing digestive and excretory physiology in *Pergamasus longicornis*. *Shaded boxes* are notional body compartments. *Grey dashed arrows* indicate common degradative metabolism. *Dotted pale grey* ‘explosion’ is the inferred ‘egestive fraction’ compartment (*asteristic* in Fig. 7 and see text). *Roman capital underlined letters* represent amounts in that compartment. *Italic underlined letters* represent proportionality or rate constants between compartments. Prey ingestion ($I_{t}$) varies episodically and irregularly with time over feeding period for total extent of 58–96 min see Bowman (1987)—starting high (presumably) then falling away. Loss ($L_{t}$) is an unknown episodic and irregular anal function over time. The gut (G) fills rapidly (complete within 10 min); switches from filling-predominating to emptying-predominating around 2 h; and, is empty by 52.5 h—Bowman (2014). Changes in the haemocoel (H) were not observed in this study (operationally assumed to be instantaneously mixed in statistical modelling). Malpighian guanine (M) begins to form at 48 h and remains for up to 10 days. The rectal vesicle (R) is crushed during gut expansion; begins to increase in size around 18 h (as the latent egestive fraction forms in the gut?); then expands as Malpighian guanine is voided into it. Fasting/starvation sets in by about 9 days—see Bowman (2014)

Whilst not so extreme as the 40–60 times scale for half-lives between gut contraction and expansion (see Bowman 2014), the disparity in scale between the input half life and output half lives for guanine of 3–7 times show that the Malpighian tubule excretory system is another (if mildly) stiff physiological system in this model mesostigmatid (Figs. 9, 10).

### Can these physiological insights be applied to field ecology?

**Fig. 11:**
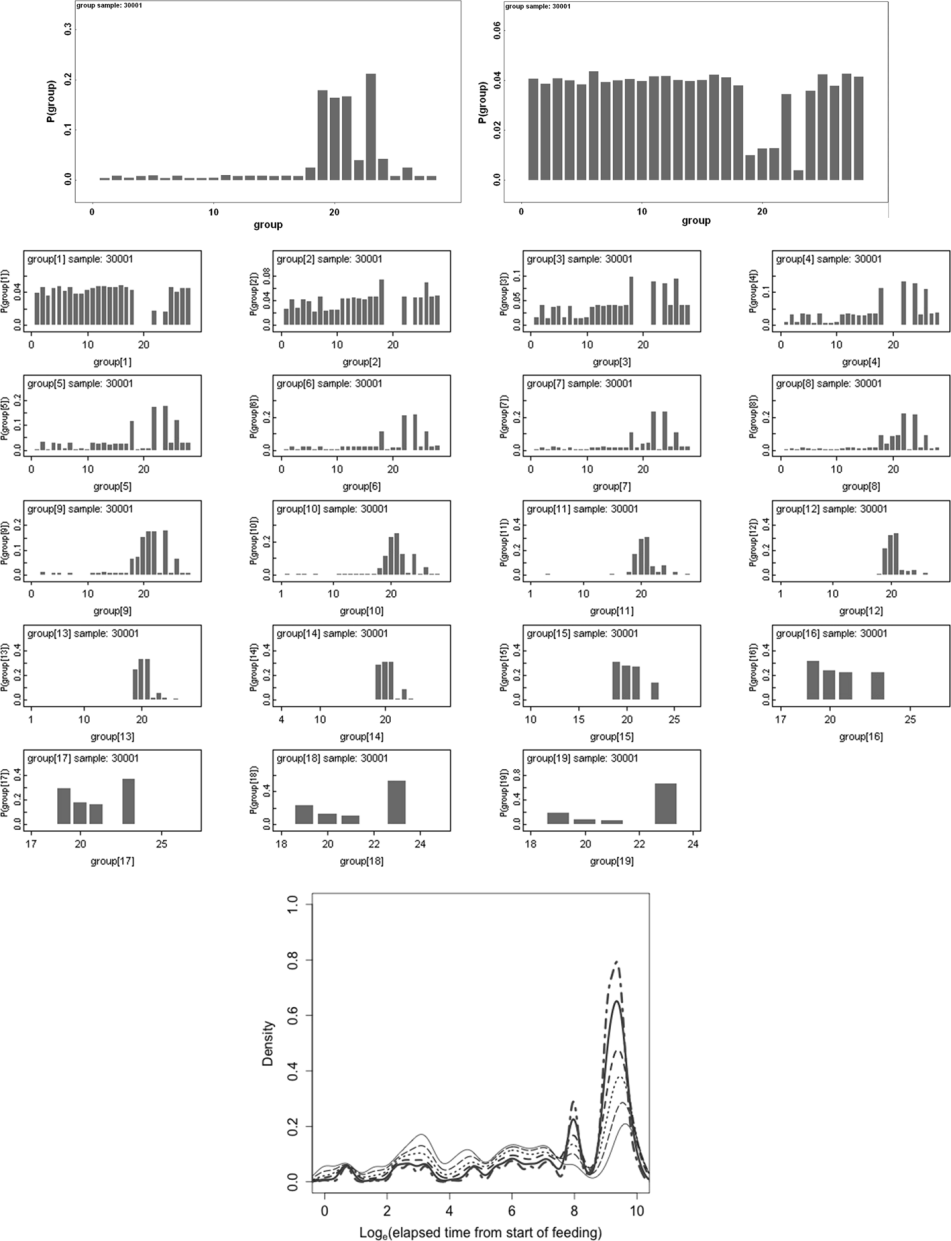
Posterior predictive distributions of likely time since the start of feeding based upon the results in this paper for use by ecologists in the field collecting *Pergamasus longicornis* and scoring their Malpighian tubule guanine. *Upper Left* Given a single mite shows Malpighian tubule guanine. *Upper Right* Given a single mite does not show Malpighian tubule guanine. Note very *small scale to left hand side* absence of guanine is not very informative. *Middle Block of small histograms* Given a number of *P. longicornis* mites (out of 20) showing Malpighian tubule guanine in a population sample. Ranges from 1 out of 20 for *top left small panel* to 19 out of 20 for *bottom right small panel*. *Lower* Nomogram of posterior predictive density for 1–6 mites out of 20 showing guanine in an ecological sample versus log$_e$ time in min after the start of feeding. 1 mite = *thin solid line*, 2 = *long dash line*, 3 = *dotted line*, 4 = *dashed line*, 5 = *solid thick line*, 6 = *bold dot-dashed line*. Note that as few as 5–6 mites scoring positive for Malpighian tubule guanine out of 20 infers a better than a 50:50 bet that the typical time from start of feeding in that population sample was about 6 days ago (i.e. around time index = 22). Any larger proportions out of 20 indicate the same peak inferred time—spanning around 2.7–7.7 days since the start of feeding

 Schütte et al. (2006) uses the carriage of accumulated white excretory material in the rectum of *Phytoseiulus persimilis* diagnostically in the field. Based upon the results found in this paper, Fig. 11 shows the posterior predictive probability distributions for the likely time from the start of feeding that can be inferred from:

- A single *P. longicornis* mite in a field sample showing Malpighian tubule guanine through its integument—*Upper Left*
- A single *P. longicornis* mite in a field sample not showing Malpighian tubule guanine—*Upper Right*
- Various numbers of mites (1–19) showing Malpighian tubule guanine in a field population sample of 20—*Middle Block*.

Included in Fig. 11 is a comparative nomogram for 1–6 mites out of 20—*Lower Centre* (other proportions or denominators can be supplied on request). Given that this mite is a bolus feeder—this latter lookup figure may be useful for soil ecologists to track typical (or expected) *P. longicornis* feeding status over seasons and geography. An irregular intermittent feeder like this mite should not have other confounding pulses of repeatedly ingested prey material being simultaneously processed.

## Conclusion

 Bowman (2014) describes three phases of digestion in *Pergamasus longicornis* during and after feeding:

1. rapid gut filling by prey fluid imbibition (with concomitant salivary processes)
2. continued gut filling by imbibition with the concentration of gut contents through fluid loss via coxal droplets
3. slow gut emptying through prey digestion.

Post digestion levels of excretory product in the Malpighian tubules are confirmed high in *P. longicornis* as predicted by Bowman (2014). However, the time course of Malpighian guanine production is not congruent with the slow digestion phase predominating after 120 min from the start of feeding. As expected, guanine crystals are not apparent in the Malpighian tubules during the early ingestion/coxal droplet phase of prey feeding (up to 1.5 h after the start of feeding). Malpighian mechanisms cannot thus be the homeostatic mechanism Bowman (2014) sought for the early stasis in gut size nor during the second later phase of feeding as there is no evidence in this study of Malpighian tubule expansion to eliminate fluids. Size changes in the Malpighian tubule system do not suggest that this system is osmoregulatory.

Malpighian guanine does not appear to be produced during the early digestive phase up to when the mite stops feeding either (i.e. during the ‘anal dabbing’ phase <1.5 h from the start of feeding—Bowman 1984). Surprisingly however, Malpighian guanine is not produced over the *total* remaining period of slow digestion as expected—rather it appears to be confined solely only to a very late period (48 h—10 days). Peaking around 5 days from the start of feeding—much like the peak guanine excretion in argasids of 2.5–5.5 days after feeding (Hamdy 1973). Guanine production appears to originate from a catabolic physiological cascade. Malpighian tubules appear to be solely excretory.

Given that assimilation occurs, the results herein support Bowman (2014)’s schema plus a fourth tri-partite phase:

- a subsequent sub-phase of inferred ‘hidden’ catabolism—a histologically latent egestive fraction within the contracting gut shadowing contributions to Malpighian inputs,
- followed by the steady appearance late-on of Malpighian tubule excretory product (extracted unseen from the haemocoel),
- and then finally, slower faecal and excretory product physical voiding.

This latter fourth three-part phase is a ‘missing link’ between the 52.5 h for overall feeding claimed by Bowman (2014) and the one week for feeding and digestion originally claimed without substantiation by Bowman (1984). A complete cycle of feeding, digestion and excretion is in fact more like 9 days long. Hunger/starvation probably begins around 10 days after the commencement of feeding. Up to 15 days may be needed to clear the idiosoma of excretory material.

Figure 10 illustrates a proposed summary scheme and first-order linear differential equation basis to understanding and quantitatively modelling this mite’s nutritive physiology, together with where the suggested latent egestive fraction might arise. Careful intensive examination of the lumenal contents of the gut during feeding and digestion remains to be done, including perhaps direct biochemical assay of the appearance and disappearance of prey material in the gut, and the appearance of excretory guanine crystals in the Malpighian tubules of *P. longicornis*. Characterising the scale of gut lumenal clearance of prey material could also be useful.

The relative physical sizes of digestive and excretory organs over time in *P. longicornis* are constrained by the mite living in a ‘suit of armour’ of finite extent. One organ swelling induces another to be compressed. This physicality drives most of the phenomena observed in this study. However; how the Malpighian tubules are emptied of Malpighian tubule material themselves when the gut is still contracting; or how the rectal vesicle at other times is emptied of Malpighian tubule material or any egested gut materials in this soil predatory mite remains to be critically demonstrated.
